# A non-invasive magnetic resonance imaging approach for assessment of real-time microcirculation dynamics

**DOI:** 10.1038/s41598-017-06983-6

**Published:** 2017-08-07

**Authors:** Tameshwar Ganesh, Marvin Estrada, Herman Yeger, James Duffin, Hai-Ling Margaret Cheng

**Affiliations:** 1 Leslie Dan Faculty of Pharmacy, University of Toronto, Toronto, Canada; 2 Translational Biology & Engineering Program, Ted Rogers Centre for Heart Research, Toronto, Canada; 3 Lab Animal Services, Hospital for Sick Children, Toronto, Canada; 4 Developmental & Stem Cell Biology, Hospital for Sick Children Research Institute, Toronto, Canada; 5 Department of Laboratory Medicine & Pathobiology, University of Toronto, Toronto, Canada; 6 Department of Anesthesia, University of Toronto, Toronto, Canada; 7 Translational Medicine, Hospital for Sick Children Research Institute, Toronto, Canada; 8 The Edward S. Rogers Sr. Department of Electrical & Computer Engineering, University of Toronto, Toronto, Canada; 9 Institute of Biomaterials & Biomedical Engineering, University of Toronto, Toronto, Canada

## Abstract

We present a novel, non-invasive magnetic resonance imaging (MRI) technique to assess real-time dynamic vasomodulation of the microvascular bed. Unlike existing perfusion imaging techniques, our method is sensitive only to blood volume and not flow velocity. Using graded gas challenges and a long-life, blood-pool *T*_1_-reducing agent gadofosveset, we can sensitively assess microvascular volume response in the liver, kidney cortex, and paraspinal muscle to vasoactive stimuli (i.e. hypercapnia, hypoxia, and hypercapnic hypoxia). Healthy adult rats were imaged on a 3 Tesla scanner and cycled through 10-minute gas intervals to elicit vasoconstriction followed by vasodilatation. Quantitative *T*_1_ relaxation time mapping was performed dynamically; heart rate and blood oxygen saturation were continuously monitored. Laser Doppler perfusion measurements confirmed MRI findings: dynamic changes in *T*_1_ corresponded with perfusion changes to graded gas challenges. Our new technique uncovered differential microvascular response to gas stimuli in different organs: for example, mild hypercapnia vasodilates the kidney cortex but constricts muscle vasculature. Finally, we present a gas challenge protocol that produces a consistent vasoactive response and can be used to assess vasomodulatory capacity. Our imaging approach to monitor real-time vasomodulation may be extended to other imaging modalities and is valuable for investigating diseases where microvascular health is compromised.

## Introduction

Measuring pathological alteration in local blood volume and flow is a diagnostic imaging mainstay best known for cancer detection and diagnosis. Imaging tumor microvascular changes, namely, increased vascularity and poor perfusion, is achieved using contrast-enhanced techniques, where a tracer is administered intravenously and its accumulation and distribution determined to distinguish the tumor from surrounding normal tissue. Contrast-enhanced imaging also benefits the diagnosis of a variety of other medical conditions that involve changes in local blood perfusion, such as myocardial infarction^1^, stroke^2^, Alzheimer’s^3, 4^, and diabetes^5^. The underlying microvascular pathology differs in these conditions (e.g. upstream obstruction, changes in the capillary wall ultrastructure^2, 3, 6–8^, etc.) from the oncology context, but the utility of contrast-enhanced imaging remains since the changes in local blood volume and/or flow are permanent. For this reason, conventional contrast-enhanced imaging suffices even though it provides only a *static* snapshot of microvascular function.

A completely different concept to conventional static imaging of microvessel function is imaging their *functional dynamics*. That is, we temporally monitor how blood vessels respond to changing metabolic and physical demands, which cannot be captured with a static measurement at a single time-point. This diagnostic capability is important when ultrastructural or neuronal deficits that compromise vasomodulation are present, but where overt changes in blood volume or flow have not yet manifested. A simple example of this concept is the cardiac stress test, where perfusion differences between normal and ischemic myocardium are highlighted when the subject performs exercise, whereas at rest there may be no perfusion differences detectable. By employing two time points for comparison, before and after exercise, the stress test allows us to assess the capacity for *response*.

The ideal assessment of microvessel functional dynamics involves repeated measurements at multiple time-points. There are two classes of techniques currently available to achieve this goal: focal and whole organ assessment. Focal techniques are restricted to skin applications and include: laser Doppler fluxmetry of blood flow^9, 10^, transcutaneous oxygen tension^11^, and iontophoresis^12^, which delivers vasoactive agents sub-dermally and measures blood flow using laser-based methods^13^. Whole organ approaches address the limited penetration depth associated with focal techniques. Examples in this category include: electron-beam computed tomography (CT)^14^, magnetic resonance imaging (MRI)^15^, and intravascular Doppler ultrasound^16^, which are used to assess blood volume changes in response to vasoactive agents such as acethylcholine and nitroprusside. However, these methods are fraught with several significant limitations. For example, vasoactive agents have been shown to generate large intra-subject variability^17^, and prolonged and/or repeated infusions may lead to tachyphylaxis^18^. To date, a non-invasive 3D technique does not exist for the assessment of microvascular functional dynamics in whole organs deep in the body.

In this paper, we propose a novel 3D MRI-based technique for the assessment of microvascular functional dynamics by measuring in near real-time blood volume changes in whole organs *in vivo*, addressing the main limitations of existing methods. Our technique employs a vasoactive stimulus in the form of air with altered levels of carbon dioxide (CO_2_) and oxygen (O_2_). Inhaling elevated CO_2_ levels (hypercapnia) and lowered O_2_ levels (hypoxia) stimulates changes in blood volume in normal physiology, and measuring such changes can assess vascular tone modulatory capacity in response to stress. Unlike pharmacological agents, the use of CO_2_ and O_2_ is not associated with adverse side effects or with prolonged monitoring time^19^. In fact, moderate hypercapnia with end-tidal CO_2_ (P_ETCO2_) ranging between 40 and 50 mmHg^20^ and moderate hypoxia with end-tidal O_2_ (P_ETO2_) up to 45 mmHg^21–23^ have been shown to be well tolerated in humans, and at these levels, blood volume can be modulated in extracranial tissues^24–28^. A second critical aspect of our approach is the use of a stable, long-life blood-pool agent (gadofosveset) to allow accurate assessment of blood volume changes that occur dynamically over the course of minutes. The proposed MRI technique demonstrates, for the first time, a new surgically non-invasive 3D imaging capability to visualize and assess microvascular response, specifically microvascular blood volume changes, to transient gas stimuli in abdominal organs, using a time scale consistent with physiological dynamics.

## Methods

### Controlled Gas Delivery Setup and Animal Monitoring

A controlled gas mixing circuit was built for the purpose of delivering graded levels of CO_2_ and O_2_, and animals were mechanically ventilated to allow control over the rate and depth of breathing. The system consisted of a computer-controlled GSM-3 gas mixer (CWE Inc., Ardmore, PA, USA) to blend the desired gas mixture at a constant total flow rate of 5 L/min. The resulting gas mixture was fed first into an isoflurane vaporiser (2 L/min) and then into an MRI compatible ventilator (MRI-1 Ventilator; CWE, Ardmore, PA, USA). Vital signs (heart rate, blood oxygen saturation) of the animal were monitored and recorded using a rodent oximeter and physiological monitor (MouseOX Plus; STARR Life Sciences) mounted on the hindpaw. These physiological measurements were taken in real-time to ensure we could monitor the animal’s status and provide an indication on what gas challenge levels were safe.

### Animal Preparation

This study was approved by the Lab Animal Services animal care committee at the Hospital for Sick Children (protocol #22500), and all procedures were conducted in accordance with the Canadian Council on Animal Care. Female adult Sprague Dawley rats (*N* = 21) (Charles River Laboratories) weighing 250–300 g were used for this study. The animal was anesthetized on 5% isoflurane in room air (2 L/min flow rate of isoflurane, Forene, Abbott Labs, Baar, Switzerland) and then administered a series of gas challenges as previously described by Ganesh *et al*.^24^. Briefly, they were first anesthetized in an induction chamber and then placed supine on an inclined plane and intubated with a 14-gauge angiocatheter. For the fourteen animals on which MRI was performed, the animal was transferred supine immediately after intubation to the imaging coil, resting on a water blanket maintained at 38 °C (HTP-1500, Adroit Medical Systems, Loudon, TN). The endotracheal tube was connected to the flexible tubing from the gas delivery system, and the animal was ventilated at a rate of 65 to 70 breaths per minute, adjusted to maintain arterial blood gases within physiological limits. The chest region was observed for a rise-and-fall motion consistent with proper respiration. The sensors of the oximeter were then clipped on the hindpaw. The animal was maintained for the duration of the imaging experiment on 2% isoflurane in room air or different gas mixtures at a flow rate of 1 L/min.

### *In-vivo* MRI

Imaging was performed on a 3-Tesla clinical scanner (Achieva 3.0 T TX, Philips Medical Systems, Best, The Netherlands), using an 8-channel wrist coil for signal detection. Rats were placed supine, feet first within the coil. The heart rate and blood oxygen saturation were continuously monitored using the pulse oximeter. Localizer scans were first acquired to determine placement of the imaging volume on the thoracic-abdominal region. Coronal imaging slices were positioned to encompass the kidney, liver, and paraspinal muscle.

To determine the time interval over which contrast enhancement in blood remained fairly stable, *T*_1_-maps were acquired every 20 minutes after administering gadofosveset (trade names Vasovist, Ablavar) via the tail vein at a dose of 0.3 mmol/kg. Two animals were used to determine the contrast profile while they were maintained on room air (no gas challenge). *T*_1_ mapping was performed using a variable flip-angle approach^29^ on a 3D spoiled-gradient echo acquisition: repeated at flip angles of 2°, 10°, and 20°; other parameters were repetition time (TR) = 6.13 ms, echo time (TE) = 3.2 ms, number of signal averages (NSA) = 8, 100 mm field-of-view (FOV), twenty-two 1-mm thick slices, and 0.6 × 0.6 mm in-plane resolution, acquisition time ~6 min.

Gas challenge experiments were performed by placing animals initially on room air and acquiring a baseline anatomical 3D *T*_1_-weighted spoiled-gradient echo sequence with fat suppression: TR = 3.73 ms, TE = 1.85 ms, flip angle = 20°, NSA = 3, 100 mm FOV, ten 3-mm thick slices, and 0.7 × 0.7 mm in-plane resolution, acquisition time = 31.4 s. Pre-injection *T*_1_ mapping was again performed to enable calculation of post-injection *T*_1_ relaxation times via the gradient echo signal equation^30^. Gadofosveset (0.3 mmol/kg) was then administered via the tail vein, and 8–12 minute-duration gas challenges beginning with normoxia (21% O_2_) were sequentially applied. Four different types of challenge were studied: extreme hypercapnia (20% CO_2_), mild hypercapnia (2–5% CO_2_), hypoxia (12% O_2_), and hypercapnic hypoxia (20% CO_2_ + 12%O_2_). Four animals were used to study each level of gas stimulus (exceptions are indicated in figure captions). The 3D *T*_1_-weighted sequence was repeated every 2 minutes throughout the session for a total duration of at most 40 minutes after contrast administration.

### Data Analysis

MRI data was transferred to an independent workstation for data analysis using Matlab (v.8.3) (MathWorks, Natick, MA). *T*_1_-maps were computed as described previously^29^. In the absence of gas challenge, changes in post-contrast *T*_1_ were determined from *T*_1_-maps in regions of interest (ROIs) outlined in the kidney cortex, liver, and paraspinal muscle and in the descending aorta close to the bifurcation to the iliacs to minimize flow-related effects for blood *T*_1_ quantification. To study microvascular response to gas challenge, *T*_1_ relaxation times were calculated from the post-contrast signal intensity and pre-contrast *T*_1_ via the gradient echo signal equation. The mean *T*_1_ relaxation time was calculated at all time-points across different gas challenge episodes in ROIs encompassing the liver parenchyma, kidney cortex, and paraspinal muscle at their largest cross-section.

### Statistical Analysis

Changes in the mean *T*_1_ relaxation time were then analysed to determine if significant changes from baseline (normoxia) occurred and if gas challenge-specific patterns existed in different organs. Changes in *T*_1_ were investigated using one-way analysis of variance (ANOVA), with the main effect being the gas challenge. Post-hoc Tukey-Kramer testing was then performed at the 95% confidence level. Significance is reported at a *p*-value of 5% unless otherwise stated.

Heart rate and blood oxygen saturation measurements from the mouse oximeter were also compared using ANOVA. A one-way analysis was performed, with the main effect being the gas challenge. Significance is reported at a *p*-value of 5%.

### Laser Doppler Perfusion Measurements

Real-time measurements of relative tissue perfusion were obtained in the liver and kidney cortex to validate the changes observed on MRI. Seven rats were used on four different gas inhalation protocols. The OxyFlo (Oxford Optronix Ltd., Oxford, UK) fiber-optic system was used to provide minimally invasive (using fiber optic probes 250–450 microns in diameter) real-time monitoring of tissue perfusion. The OxyFlo system uses laser Doppler flowmetry to provide continuous monitoring of blood flow in relative perfusion units (BPU). The BPU is useful for assessing relative perfusion changes in an organ but should not be used to compare different tissue types, which tend to differ in their optical properties.

To perform invasive perfusion measurements, a laparotomy was performed to expose the liver and kidney while the animal was anesthetized on isoflurane, and the exposed organs were covered with wet gauze to maintain hydration. One Oxyflo channel was used to monitor perfusion from a probe inserted between liver lobes, and a second channel was used to monitor perfusion from a probe inserted under the renal capsule to measure perfusion in the kidney cortex. These probe placement strategies were chosen to avoid puncturing the liver and causing bleeding and to ensure kidney measurements were made in the cortex and not in the medulla. To mitigate effects of respiratory motion on the liver, the probes were sutured to the skin such that the probes would follow the motion of the respiratory cycle.

### Data Availability Statement

The datasets generated during and/or analysed during the current study are available from the corresponding author on reasonable request.

## Results

**Figure 1:**
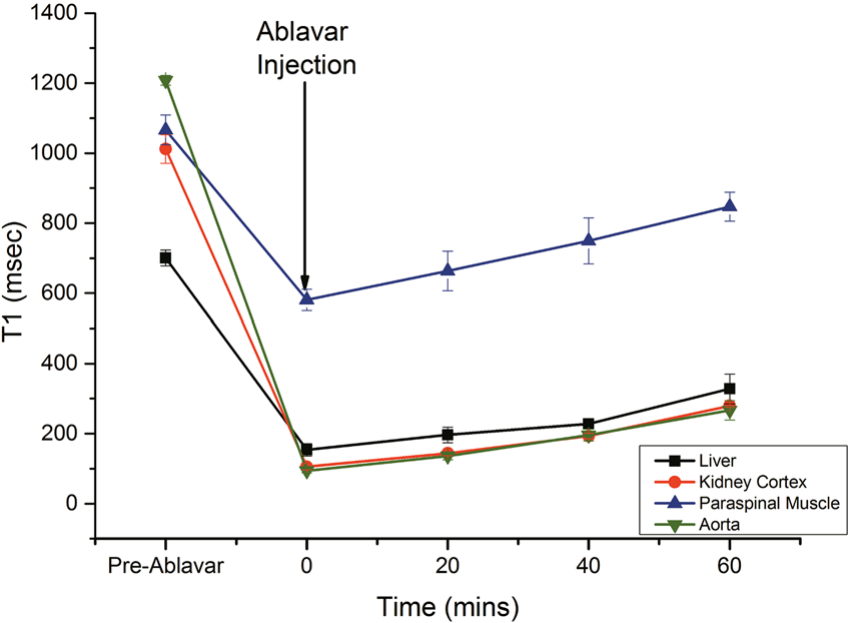
Stability of the blood-pool agent gadofosveset in the microvascular bed. Temporal evolution of mean *T*_1_ relaxation times and standard error in the aorta, liver, kidney, and paraspinal muscle post-administration of gadofosveset in the absence of gas challenge stimuli (*N* = 2).

Figure 1 shows the time-course change in *T*_1_ relaxation time post-gadofosveset injection in blood, liver, kidney cortex, and paraspinal muscle in the absence of gas challenge. Although gadofosveset has a slower renal elimination rate than non-blood-pool agents, it is clear from Fig. 1 that *T*_1_ increases steadily in all tissue types as the *T*_1_-reducing agent is slowly excreted from the body. We chose to limit our “stable” window of investigation to the first 40 minutes post-injection where the *T*_1_ fluctuation in blood was less than double the baseline *T*_1_ value.

**Figure 2:**
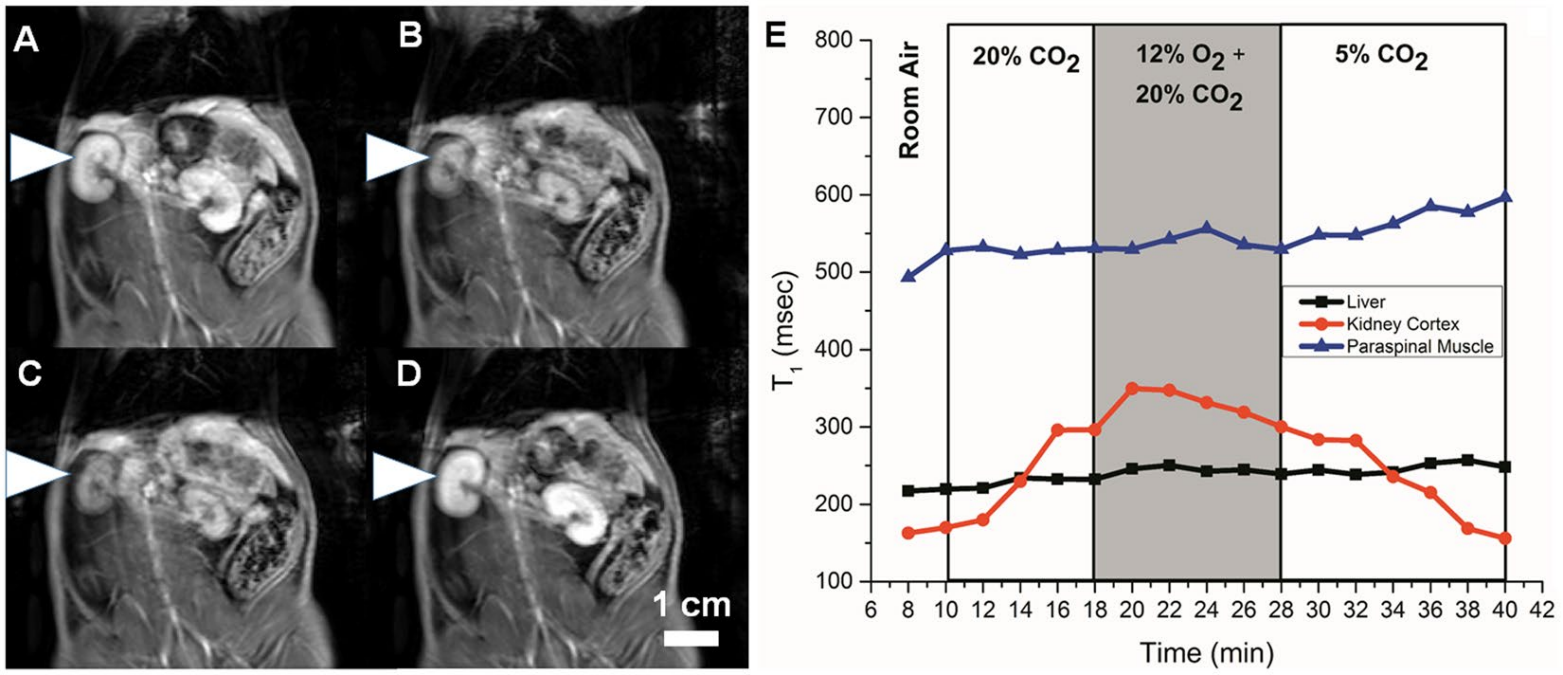
Dynamic MR imaging of hypercapnia- and hypercapnic hypoxia-mediated vasomotion. The kidney is shown on *T*_1_-weighted images acquired at the end of successive 8–12 minute gas challenges in a rat subjected to the following sequence: normoxia (**A**) → hypercapnia (20% CO_2_) (**B**) → hypercapnic hypoxia (20% CO_2_ + 12% O_2_) (**C**) → hypercapnia (5% CO_2_) (**D**). Corresponding temporal evolution of changes in *T*_1_ relaxation times in the liver, kidney, and paraspinal muscle (**E**).

**Figure 3:**
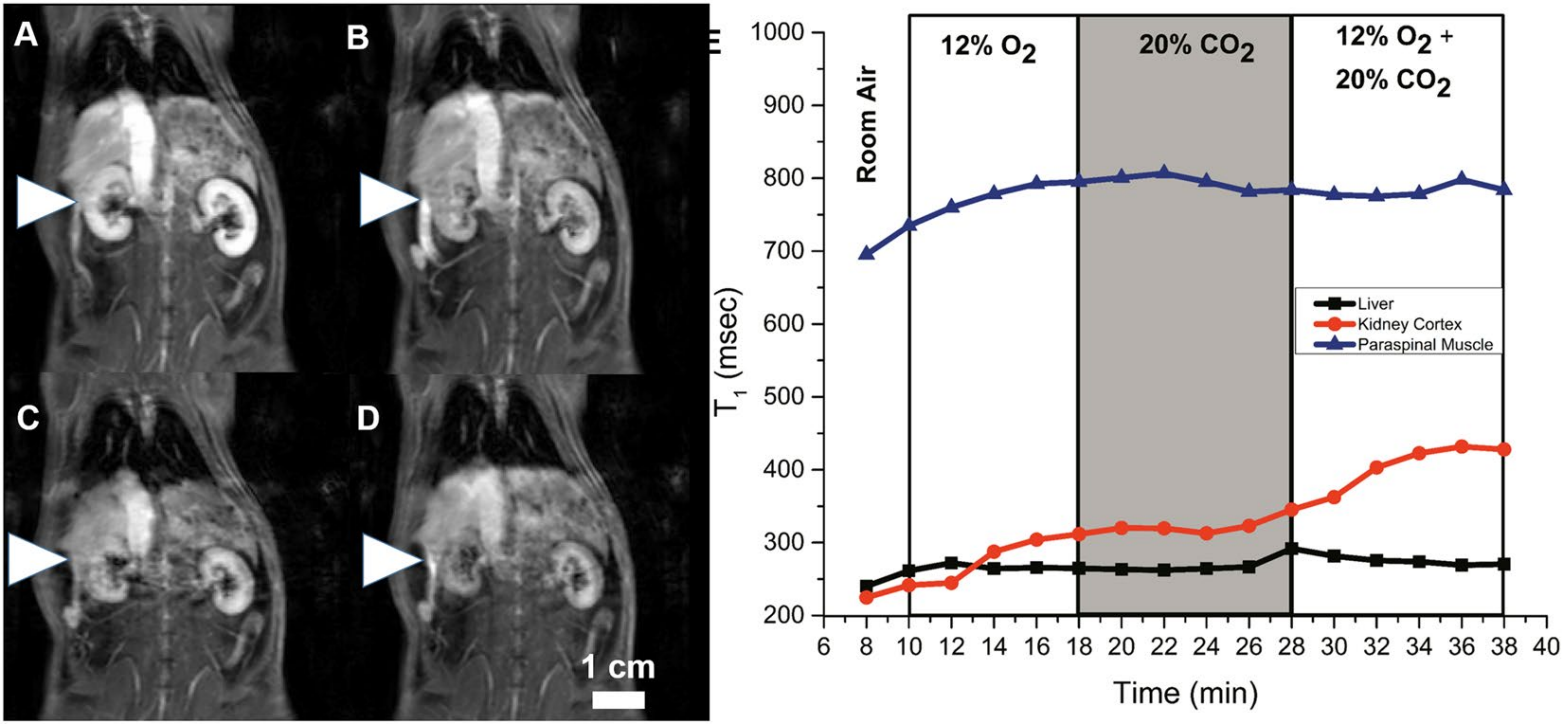
Dynamic MR imaging of differential microvascular response to hypercapnia and hypoxia. The kidney is shown on *T*_1_-weighted images acquired at the end of successive 8–12 minute gas challenges in a rat subjected to the following sequence: normoxia (**A**) → hypoxia (12% O_2_) (**B**) → hypercapnia (20% CO_2_) (**C**) → hypoxic hypercapnia (12% O_2_ + 20% CO_2_) (**D**). Corresponding temporal evolution of changes in *T*_1_ relaxation times in the liver, kidney, and paraspinal muscle (**E**).

**Figure 4:**
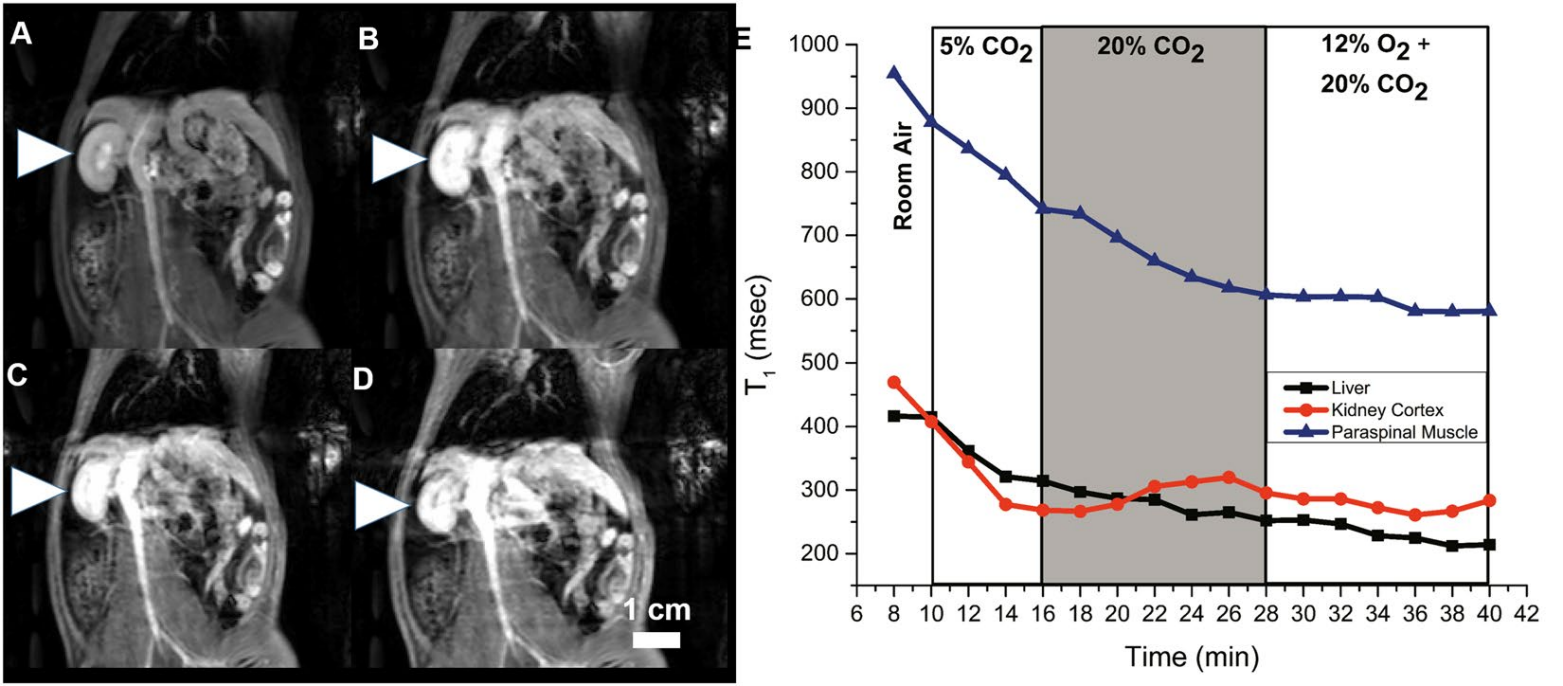
Dynamic MR imaging of blunting effect of mild hypercapnia-mediated vasodilatation on subsequent vasoconstriction capacity. The kidney is shown on *T*_1_-weighted images acquired at the end of successive 8–12 minute gas challenges in a rat subjected to the following sequence: normoxia (**A**) → mild hypercapnia (5% CO_2_) (**B**) → severe hypercapnia (20% CO_2_) (**C**) → hypoxic hypercapnia (12% O_2_ + 20% CO_2_) (**D**). Corresponding temporal evolution of changes in *T*_1_ relaxation times in the liver, kidney, and paraspinal muscle (**E**).

Examples of changes on *T*_1_-weighted images and in *T*_1_ relaxation times for three gas challenge protocols are shown in Figs 2–4. Note that bright signal on *T*_1_-weighted images is associated with a low *T*_1_ relaxation time. Images shown were acquired at the end of the gas challenges indicated (subfigures A to D), and the corresponding time-course change in *T*_1_ for each protocol is also shown (subfigure E). Figure 2 illustrates our ability to visualize the evolving vasoconstriction effected by hypercapnia and hypercapnic hypoxia, which is particularly pronounced in the kidney cortex where the signal decreased dramatically upon hypercapnia and was further sustained with hypercapnic hypoxia. Mild hypercapnia (5% CO2) brought about elevated signal consistent with vasodilatation, as expected. Figure 3 illustrates the differential vasoconstrictory effects of hypoxia and hypercapnia, and it is seen that hypercapnia induces greater vasoconstriction than does hypoxia, with the largest change observed for both stimuli combined. Figure 4 illustrates the blunting effect of mild hypercapnia on successive severe hypercapnic stimuli. Except for the scenario shown Fig. 4, changes were much milder in the liver and paraspinal muscle.

**Figure 5:**
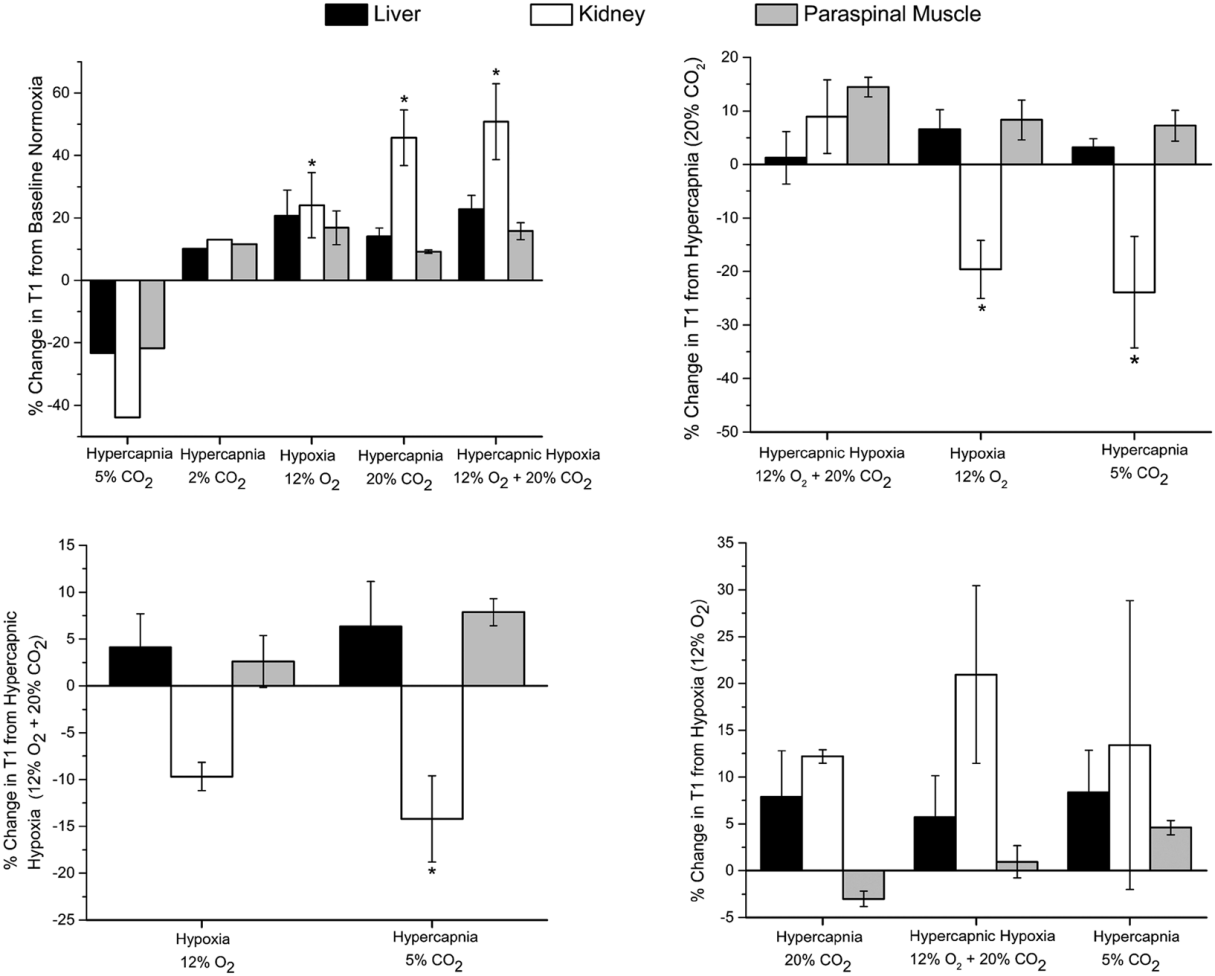
MRI response to gas challenges. Mean changes in *T*_1_ relaxation times for different gas challenge regimes averaged across all animals (*N* = 4 per group) and standard error are shown for the liver, kidney cortex, and paraspinal muscle. Exception is 2% CO_2_, which was studied in a single animal. Significant differences from baseline challenges are indicated (*P < 0.05).

Figure 5 summarizes across all animals the changes in *T*_1_ relaxation times for different gas challenge transitions, starting from different baseline gases. An increase in *T*_1_ was observed in all tissues upon transition from normal air to severe hypercapnia or hypoxia or both – kidney cortex (24% to 50% increase), liver (15% to 22% increase), and paraspinal muscle (9% to 16% increase). The trends in *T*_1_ are consistent with greater vasoconstriction in going from hypoxia to hypercapnia and finally hypercapnic hypoxia. Only 2% CO_2_ showed no significant change from baseline air, but 5% CO_2_ produced a marked decrease in *T*_1_ suggestive of vasodilatation. When starting from either a hypercapnic (20% CO_2_) or hypercapnic hypoxic baseline, a trend toward decreased *T*_1_ was observed for 12% O_2_ and 5% CO_2_ in the kidney cortex, suggestive of vasodilation that is expected to occur. Transitions from a hypoxic baseline produced changes consistent with the lower vasoconstrictory effect of hypoxia compared to hypercapnia.

**Figure 6:**
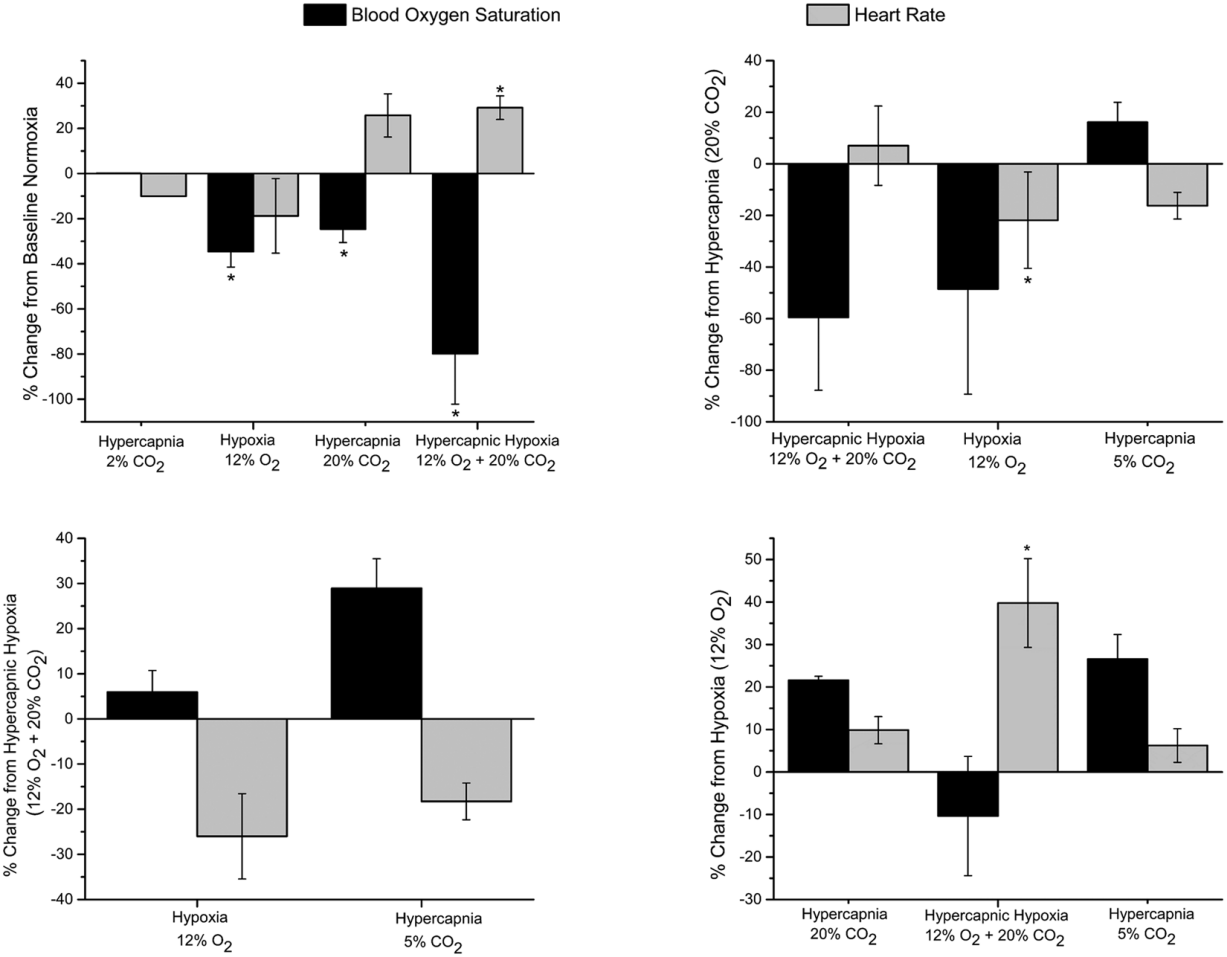
Heart rate and blood oxygen saturation response to gas challenges. Mean changes in pulse oximeter measured blood oxygen saturation and heart rate for different gas challenge regimes averaged across all animals (*N* = 4 per group) and standard error are shown for the liver, kidney cortex, and paraspinal muscle. Exception is 2% CO_2_, which was studied in a single animal. Significant differences from baseline challenges are indicated (*P < 0.05).

Pulse oximeter measurements of heart rate and blood oxygen saturation for all gas challenge transitions are shown in Fig. 6. Starting from normal air, heart rate increased for 20% CO_2_ (25% increase) and hypercapnic hypoxia (30% increase) but decreased for hypoxia (19% decrease) and mild hypercapnia (10% decrease). Blood oxygen saturation decreased for all hypercapnic and hypoxic challenges, except for 2% CO_2_. Starting from either 20% CO_2_ or a hypercapnic hypoxic baseline, heart rate decreased only upon transitioning to less stressful challenges (hypoxia and 5% CO_2_), and blood oxygen increased only on transitioning to 5% CO_2_. Starting from a hypoxic baseline, both heart rate and blood oxygen saturation increased when moving to strictly hypercapnic challenges. Significant increases in heart rate were obtained on transitioning from an isocapnic baseline to hypercapnic hypoxia.

**Figure 7:**
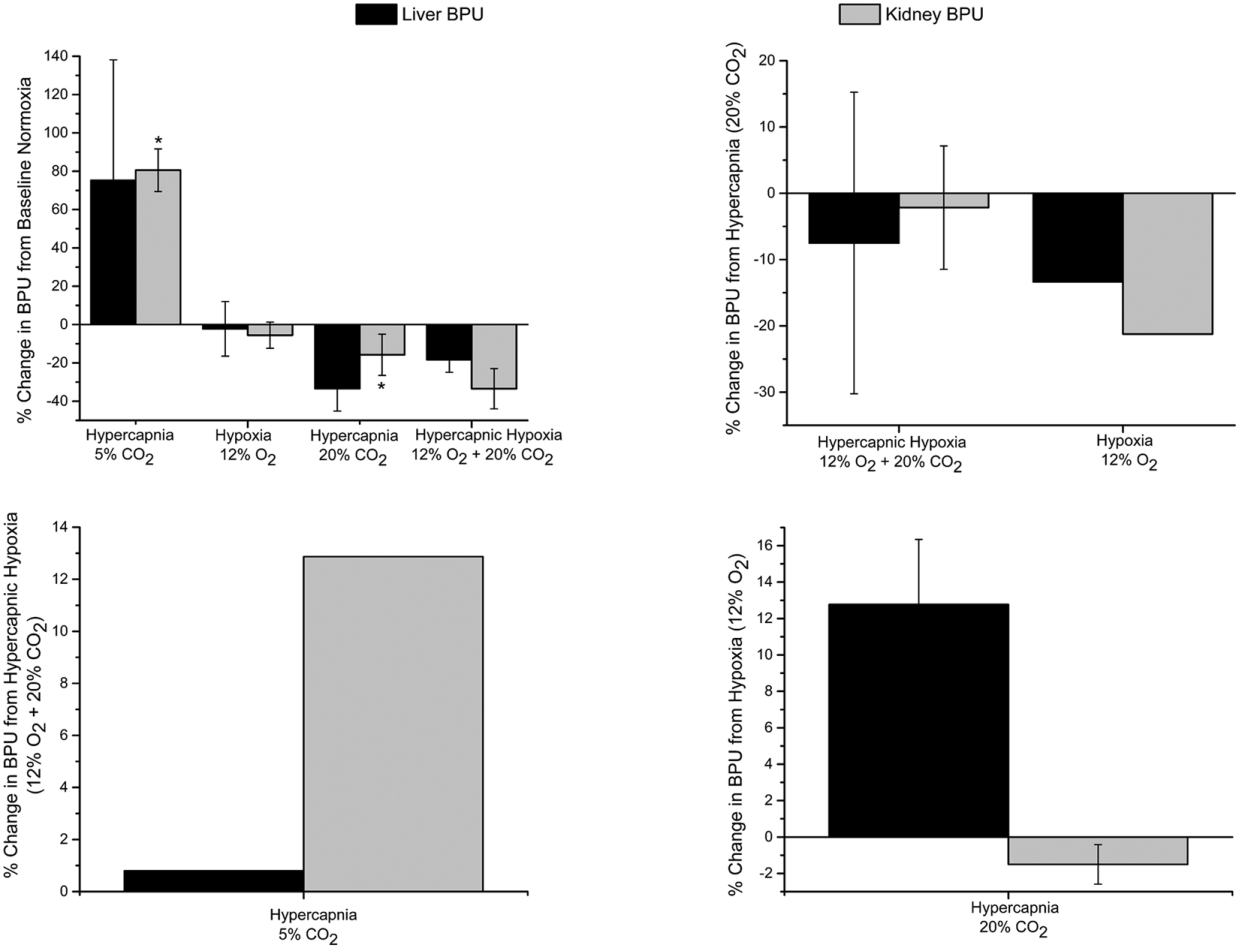
Laser Doppler perfusion response to gas challenges. Mean changes in blood perfusion units in the liver and kidney cortex for different gas challenge regimes averaged across all animals (*N* = 5 per group) and standard error are shown for the liver and kidney cortex. Exceptions are 5% CO_2_ and 12% O_2_, which were studied in a single animal. Significant differences from baseline challenges are indicated (*P < 0.05).

Figure 7 illustrates the relative change in laser Doppler-measured blood perfusion in the liver and kidney cortex for different gas challenges, again starting from different baseline gases. Perfusion increased substantially in both organs on transition from normal air to mild hypercapnia but decreased for hypoxic and more extreme hypercapnic challenges. In general, the magnitude of perfusion changes in the liver was smaller compared to the kidney cortex, a trend consistent with MRI observations.

**Figure 8:**
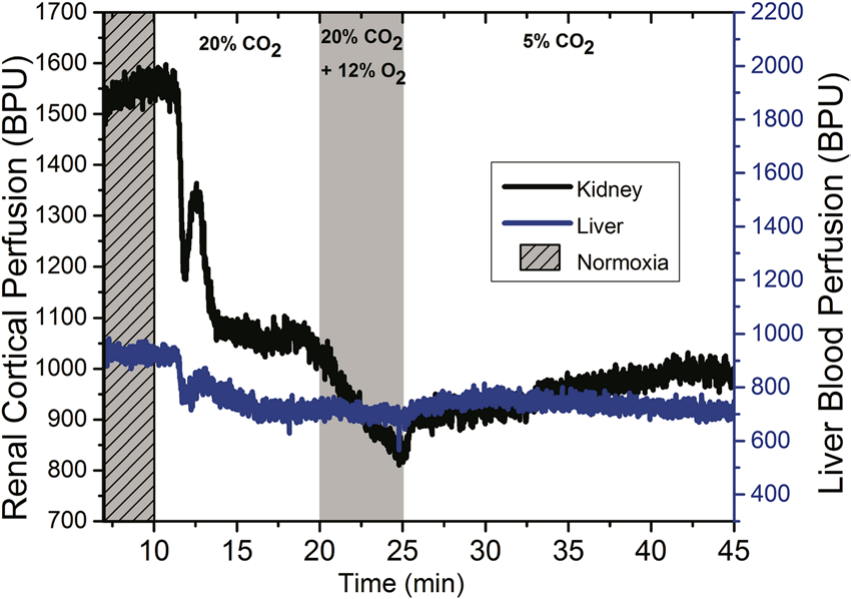
Real-time tissue perfusion response in the liver and kidney cortex. Dynamic laser Doppler perfusion recordings are shown for the following gas sequence: normoxia→ hypercapnia (20% CO_2_) → hypercapnic hypoxia (20% CO_2_ + 12% O_2_) → hypercapnia (5% CO_2_). Units are in relative blood perfusion units (BPU).

A sample real-time perfusion recording obtained from laser Doppler measurements is shown in Fig. 8 for both the kidney cortex and the liver. The decrease in perfusion observed on transition to 20% CO_2_ and further decrease on transition to hypercapnic hypoxia, is analogous to similar signal decreases on MRI (see Fig. 2). Likewise, mild increases in perfusion on the transition to 5% CO_2_ correlates with increased signal on MRI. These gold-standard perfusion measurements support the interpretation of increasing and decreasing signal intensity as indicators of vasodilatation and vasoconstriction, respectively.

## Discussion

Microvascular dysfunction (i.e. compromised capacity to adjust microvascular blood volume) is at the heart of a variety of pathologies ranging from stroke^31^ to diabetes^32^. The approach of most current diagnostics is measurement of blood volume changes pre- and post-administration of pharmacological agents that alter vascular tone. Most applications to date have been limited primarily to the skin, but some groups have attempted to probe deeper organs using non-invasive techniques such as CT^33^, positron emission tomography (PET)^34^, ultrasound^35^, and MRI. These methods are also limited in terms of radiation burden (CT and PET), low spatial resolution (PET and ultrasound^36^), and reliance on pharmacological interventions that carry significant risks of side effects. The use of MRI to assess microvascular volume dynamics has been limited, with the earliest MRI attempts using solely a non-pharmacological stimulus, hypercapnia, to modulate vasoactive tone^37^, and subsequent studies adding in an extracellular contrast agent in an attempt to monitor changes in blood volume^25^. Yet, due to the short life time of the contrast agent in blood, accurate assessment of vasomodulation was not possible.

In this study, we present a non-invasive technology for 3D assessment of dynamic response in microvascular blood volume deep in the body. Our approach has a unique combination of critically important novel features. The first feature is an optimized gas challenge protocol that will *consistently* elicit vasoconstriction followed by vasodilatation. This protocol was designed after thorough testing of the body’s physiological response to different gases and the order of delivery. The second important feature is the use of a stable, long-life blood-pool *T*_1_-reducing agent, gadofosveset, to address two important limitations of traditional vasoreactivity assessment^25^, which is confounded by blood oxygen or flow effects. By introducing contrast and using *T*_1_-weighted imaging, we sensitize the acquisition to microvascular blood volume and remove any confounding influence from blood oxygen level. Also, the relatively stable enhancement profile of a blood-pool agent versus an extravascular agent provides a constant baseline over an intended time interval; this window is essential to applying gas challenges that must be sustained long enough to produce a vasomodulatory effect and to ensuring that any signal change occurring over that interval is primarily due to changes in microvessel volume and not contrast elimination. It is important to note that while blood-pool agents have been used for quantifying *static* blood volume^38^, its application to capture vasomodulation has never been reported.

In interpreting the observed MRI responses, we shall also revisit known underlying physiological responses to gas challenges where appropriate.

Systemically, hypercapnia has a significant impact on the cardiovascular and respiratory systems. An important consequence of hypercapnia is respiratory acidosis, which reduces left ventricle contractility and stroke volume^39, 40^ but is compensated for by an increased heart rate^40^. This response explains the 25% increase in heart rate we observed on pulse oximetry for the transition from normoxia to 20% CO_2_. Elevated heart rates may also result from activation of peripheral chemoreceptors^41^ and sympathetic neural activation via increased plasma levels of both norepinephrine and epinephrine^42, 43^. Therefore, the modulation of the presynaptic muscarinic inhibition of norepinephrine release, and/or increased plasma levels of catecholamine, may be the major mechanisms of the acidosis-induced increases in heart rate. In addition to affecting the chronotropic behaviour of the heart, hypercapnia has been linked to varying degrees of vasomodulation. Mild hypercapnia mediates local vasodilatation in cerebral arteries^44^. In the kidney, mild to moderate hypercapnia results in vasoconstriction^45^, and reduced perfusion at levels above 7% CO_2_ has been linked to activation of the renin-angiotensin system^46, 47^. In the liver, hypercapnia below 7% CO_2_ has been linked to an increase in total liver blood flow via vasodilatation of mesenteric vascular system^48, 49^, whereas an increase in CO_2_ above these levels increases vascular resistance and reduces total liver blood flow^50^. In our study, we observed similar response patterns. Mild hypercapnia at 2% CO_2_ resulted in a modest increase in *T*_1_ (~11%). Severe hypercapnia (20% CO_2_) exacerbated this response, resulting in a *T*_1_ increase of 45% in the kidney cortex. This *T*_1_ increase predominantly reflects a reduced microvascular volume due to vasoconstriction, since the amount of blood-pool agent in the microvasculature is the principal contributor to *T*_1_ relaxation time. Laser Doppler perfusion experiments were in agreement with MRI, showing increased perfusion for mild hypercapnia and decreased perfusion for extreme hypercapnia.

Hypoxia-mediated vasomodulation is much less characterized compared to hypercapnia. Systemic hypoxia is known to stimulate peripheral chemoreceptors that results in increased sympathetic outflow^51^ and vasoconstriction in the renal, splanchnic, liver, and skeletal muscle vascular beds^52–55^. Furthermore, hypoxic stimulation of carotid chemoreceptors during controlled ventilation has negative sympathetically mediated chronotropic and inotropic effects that result in bradycardia^56, 57^. In the present study, hypoxia reduced heart rate by 19%, consistent with expected physiological response. Also consistent with expected vasoconstriction is increased *T*_1_ observed in the kidney, liver, and the paraspinal muscle, which was confirmed by laser Doppler measurement. One interesting observation in our study was increased signal in the kidney cortex when transitioning from hypercapnic baselines to hypoxia. This differential response to hypoxia may be attributed to its biphasic behaviour wherein it mediates the release of endogenous vasodilator that counters the vasoconstrictor effect of increased sympathetic activity^58, 59^.

The final gas challenge examined in this study was hypercapnic hypoxia. This stimulus produced the greatest increase in heart rate (30%), the largest increase in *T*_1_ relaxation time, and the largest decrease on laser Doppler perfusion measurements. Hypercapnic hypoxia-mediated elevation in heart rate has been attributed to elevated plasma cathecholamine levels in response to the gas challenge^60^. The *T*_1_ signal reduction (or increased *T*_1_) observed is indicative of reduced microvascular volume, which was confirmed on laser Doppler perfusion measurements. Literature attributes this vasoconstriction to increased renal sympathetic nerve activity^61, 62^ and increased muscle sympathetic nerve activity^63^. The only literature inconsistency is a study by Davidson *et al*.^60^, who reported increased hepatic blood flow in response to hypercapnic hypoxia. In contrast, we observed reduced liver perfusion, perhaps because we employed a higher level of hypercapnic hypoxia (20% CO_2_ + 12% O_2_) compared to Davidson *et al*. (12% CO_2_ + 10% O_2_).

In addition to developing a new non-invasive technique to measure modulation of microvessel tone dynamically, a secondary objective of this study was to investigate what sequences of gas challenges could effectively assess microvascular function or dysfunction. Not only must we be able to increase or decrease microvascular volume and then return it to original baseline levels, but we must ensure that the order of gas challenges does not inhibit dynamic response. For instance, Zanzinger *et al*.^64^ showed that nitric oxide, one of the main mediators of hypercapnia-mediated vasodilation, actually inhibits sympathetic vasoconstriction. We also observed this phenomenon, where beginning the protocol with mild hypercapnia at 2% or 5% CO_2_ blunted vasoconstriction on subsequent hypercapnic and/or hypoxia stimuli (Fig. 4). The ideal gas challenge protocol is one that begins with hypercapnia or hypercapnic hypoxia to produce substantial vasoconstriction, ending with mild hypercapnia to mediate vasodilatation (see Figs 2 and 8).

In summary, we have shown that transitioning from normal air to extreme hypercapnia (20% CO_2_) or hypercapnic hypoxia (20% CO_2_ + 12% O_2_) produced the greatest reduction in perfusion and *T*_1_-weighted signal (and corresponding increase in *T*_1_ relaxation time). Since heart rate was elevated at the same time, reduced perfusion arose from vasoconstriction and not from reduced cardiac output, and lowered signal on MRI reflected a smaller microvascular volume. Further transitioning from hypercapnia to hypoxia did not necessarily bring about further vasoconstriction in all tissue types: while reduced perfusion was measured in both liver and kidney cortex on laser Doppler, MRI indicated decreased signal in liver but increased signal in kidney. This discrepancy may be due to poor whole organ resolution of the laser Doppler technique (more on this point later), because the renal MR response suggestive of vasodilation is supported by literature that shows hypoxia results in adenosine-mediated vasodilatation in the renal efferent arterioles, which overrides vasoconstriction mediated by sympathetic nerve response to peripheral chemoreceptor stimulation^51, 65^. Finally, transitioning from hypercapnia or hypercapnic hypoxia to mild hypercapnia significantly increased perfusion and *T*_1_-weighted signal in the kidney cortex, with little change in other organs. Since heart rate lowered at the same time, renal perfusion increase was a result of active vasodilatation. In reversing vasoconstriction, we noted that mild hypercapnia following an isocapnic hypoxic challenge had minimal vasodilatory effect.

The choice of contrast agent and validation tool (i.e. laser Doppler) deserves further discussion. It should be emphasized that our method for measuring microvessel modulation dynamics does not depend strictly on the use of gadofosveset, which may have limited clinical utility going forward since its recent removal from the European and U.S. market. In fact, any contrast agent that provides stable, long-life blood-pool characteristics would be appropriate, and this new application should be convincing incentive for the development and marketing of blood-pool agents in general. An alternative agent that can be used is gadobenate dimeglumine (MultiHance, Bracco Diagnostic, Milano, Italy), which also has partial albumin binding characteristics and has been shown to produce similar images as gadofosveset^66, 67^. The choice of laser Doppler for gold-standard perfusion measurement to validate MRI was motivated by its real-time acquisition capability and minimal invasiveness. However, as it provides only point-of-source measurements, laser Doppler cannot offer whole-organ assessment of microvascular response as can our MRI technique. Furthermore, we recognize that even this ideal validation tool is a perfusion, or blood flow, technique, and is not identical to our blood volume-sensitized MRI method. For instance, perfusion can change despite no change in blood volume if the perfusion pressure is altered.

Lastly, we would like to emphasize the importance of this contribution in its proper context. The technology presented herein provides a “real-time” approach to quantitatively assess microvascular physiological response to stress. The stressor applied, being a gas stimulus, induces a fairly rapid response time on the order of minutes, thereby necessitating measurements on the same temporal scale. With a view towards clinical translation, dynamic 3D *T*_1_-weighted acquisitions are ideal for rapidly capturing these microvascular changes with high spatial resolution. Also important to note is that we have used the term “blood volume” to indicate we sensitized our measurements to blood volume rather than blood flow, but we are not measuring absolute blood volume. Converting measured *T*_1_ to absolute blood volume would require knowledge of the hematocrit, which may change due to gas manipulation, and the potential effects of proton exchange. A final point to note is that the proposed technique is not optimized for studying gas responses in detail. There is inevitably background contrast elimination, and we determined that the first 40 minutes post-contrast was adequately “stable” to traverse the full vasoconstriction-vasodilation regime. However, because of the background drift, one should not compare response at the end of 40 minutes to much earlier timepoints in absolute terms. Instead, one should focus on the rapid *T*_1_ changes between successive gas challenges, which allows us to safely disregard effects from much more slowly varying background drifts from contrast elimination. The gas stimulus protocol shown in Fig. 2 was designed to elicit a measurable and reproducible response within the practical limits imposed by the contrast agent. For an in-depth investigation on the order of gas delivery, the effect of prolonged gas stimuli, and lag times upon return to room air, we refer the reader to our previous work^24^.

## Conclusions

We have presented a novel non-invasive method based on MRI to monitor the dynamics of microvessel response *in vivo*. To our knowledge, this is the only non-invasive technique reported to date that allows assessment of the capacity for vasomodulation in the microvascular bed in a volume of deep tissue. This technique enables much higher spatial resolution than arterial spin labeling approaches and circumvents specificity issues related to iron-oxide based methods for blood volume measurements. We have also developed an optimized gas challenge protocol that allows accurate assessment of microvascular tone control by inducing first vasoconstriction (an active process) and then vasodilatation (a relaxation process). The concepts presented herein for imaging assessment of dynamic vasomotion can be extended to other imaging modalities and will unlock the power to explore functional dynamics in a variety of disease settings.
